# Psycho-Social Effects of Pet Dog Ownership on Mentally Challenged Children

**DOI:** 10.7759/cureus.26389

**Published:** 2022-06-28

**Authors:** Sandeep Aggarwal, Roopashi Aggarwal, Manmeet Kaur Sodhi, Shallu Aggarwal

**Affiliations:** 1 Department of Pediatrics, Government Medical College, Amritsar, IND; 2 Department of Law, Om Prakash (OP) Jindal Global University, Sonipat, IND; 3 Department of Ophthalmology, District Hospital, Amritsar, IND

**Keywords:** psychosocial, ham-a, pets, mental disabilities, children

## Abstract

Aim: Mental retardation is a social stigma and children affected by this condition always require love and compassion. Pets have a positive role in human life to relieve stress and anxiety. Pets are therefore considered to be a very important aspect of psychological therapy. Those children who are suffering from mental retardation have to be given regular stress and anxiety-relieving sessions. Hence this study aims to analyze the psychosocial effects of pet dog ownership on mentally challenged children.

Methodology: A total of 112 children were included in the study and were counseled at the Department of Pediatrics, District Hospital, Amritsar. Twenty patients were lost to follow up and pet ownership materialized in 52 patients. The study was divided into 2 groups, the compliant group (n=52) and the non-compliant group (n=40). Hamilton anxiety scale (HAM-A) was applied to all the children before pet dog ownership (PRE) and after 3-6 months with a pet dog (POST). The pre and post-scores of all the children were recorded and subjected to statistical analysis.

Results: The HAM-A score before pet ownership was comparable, before pet ownership (p=.825), but after 3-6 months of pet ownership significant difference was observed between compliant and non-compliant groups (p<.001). Also, the HAM-A score in children with mild mental retardation (mild MR) and moderate mental retardation (moderate MR) was significantly less than the non-compliant group after 3-6 months. We also observed that the decrease in the anxiety levels was comparable in children who owned local breeds and foreign breeds.

Conclusion: This short-term follow-up research highlights the potential advantages of keeping a companion dog for youngsters with mental problems in terms of improving their lives. Many of these long-term gains might be attributed to lessening tensions within families.

## Introduction

Throughout the years, the use of animals in therapy, education, and care has grown significantly. It is now common knowledge that animal-assisted therapy (AAT) and activities are beneficial [1]. Scientific evidence backs up many of the claimed advantages of having a pet in one's life. Reduced loneliness and the capacity to start conversations are two benefits of owning a dog, according to research [2]. One area of HAI (human-animal interaction) study that has increased in popularity is looking at the potential influence of pet ownership (or "companion animals") on human physical and mental health. This field's observational study implies that human health and well-being may benefit from interactions and connections with companion animals, such as via processes involving the animal's attachment to or friendship with the owner [3-5].

Ownership of a pet has been linked to fewer visits to the doctor, lower blood pressure, and higher survival rates after myocardial infarction [6-8]. These advantages have been demonstrated in adults, but the advantages of having a pet have also been proven in children. Pets have also been linked to benefits similar to those gained from social support ties with family or friends in the population of youngsters. Many neurotypical youngsters name their pets as their closest relationships when asked to prioritize them, and they report expressing happiness, grief, and anger with them [9,10]. Children may obtain comfort and the sensation of unconditional love from their pets as they move through various developmental stages [11]. The present study was done to analyze the psycho-social effects of pet dog ownership on mentally challenged children.

## Materials and methods

The psychological and social impact of various things are there in human life and this can be checked by various methods of scales, parameter tests, etc. One such study was designed to evaluate the effects of pets on children. An investigation of the psychological and social impacts of pet ownership on children with mental disorders was undertaken in the pediatrics department of the district hospital in Amritsar, India. The research was carried out with the permission of the Ethics Committee and with the agreement of the children's legal guardians with the Institutional Review Board (IRB) number REF 12/03/2018. A total of 112 children were included in the study and were counselled. 20 patients were lost to follow up and the pet ownership materialized in 52 patients. The study was divided into 2 groups. The compliant group ( who owned a dog) included 52 children and the non-compliant group ( who did not own a dog) was formed of 40 children.

Informed consent was taken from the parents or guardians. Also, a qualified clinical psychologist analyzed the children's intelligence quotient (IQ) and established a diagnosis based on the International Classification of Diseases (ICD-10) criteria.

Demographic data of the subjects such as the age, gender, type of disease they are affected with, mental retardation (MR) degree of the subjects, and intelligence quotient were recorded. To keep the bias as minimum as possible, both the groups descriptively were kept near to each other. Hamilton anxiety scale (HAM-A) was applied to all the children before pet dog ownership (PRE) and after 3-6 months with a pet dog (POST). The pre and post score of all the children were recorded and subjected to statistical analysis.

As one of the first rating measures for anxiety symptoms, the HAM-A is still frequently used today in clinical and research settings. When it comes to assessing both mental and physical stress, the scale includes 14 things that each have their own symptoms, each of which may be described by the physical complaints related to anxiety. It has been predetermined that the results of the evaluation can be interpreted as follows: there is a total score range of 0-56, and a score of 17 or less indicates mild anxiety severity; a score from 18 to 24 indicates mild to moderate anxiety severity; and lastly, a score of 25 to 30 indicates a moderate to severe anxiety severity.

Statistics were used to examine the information that had been gathered and produced. To conduct the investigation, we tapped into the power of SPSS, version 23 (IBM Corp., Armonk, NY). Descriptive as well as comparative values were first gathered and then the tests were applied to the values generated. There was a chi-square test used for categorical data and either the t-test or analysis of variance (ANOVA) was used for data that was broken up into groups. A p-value of less than 0.05 was deemed significant, while a p-value of less than 0.001 was deemed extremely significant.

## Results

The baseline characteristics of the study participants are shown in Table 1.

**Table 1:** Distribution of baseline parameters in both the groups MR: mental retardation; IQ: intelligence quotient; SD: standard deviation

Basic characteristics		Compliance	Non compliance	p- value
Age	Mean	8.19±3.82	6.35±3.2	0.01*
Gender	Males	29(55.8%)	19(47.5%)	0.43
	Females	23(44.2%)	21(52.5%)	
Diagnosis	Cerebral palsy	19(35.5%)	8(20.0%)	0.19
	Epilepsy	9(17.3%)	14(35%)	
	Mental Retardation idiopathic	15(28.8(%)	12(30%)	
	Post Meningitis Sequelae	8(15.4%)	4(10%)	
	Tics	1(1.9%)	2(5%)	
MR degree	Mild( IQ 50-70)	28(53.80%)	20(50%)	0.71
	Moderate ( IQ 35-50)	24(46.20%)	20(50%)	
Siblings	0	25(48.1%)	18(45%)	0.95
	1	21(40.4%)	17(42.5%)	
	2	4(7.7%)	4(10%)	
	3	2(3.8%)	1(2.5%)	
House	Own	34(65.4%)	20(50%)	0.13
	Rent	18(34.6%)	20(50%)	
Literacy (mother)	Literate	49(94.2%)	30(75%)	0.009*
	Illiterate	3(5.8%)	10(25%)	
Literacy (father)	Literate	45(86.5%)	31(77.5%)	0.25
	Illiterate	7(13.5%)	9(22.5%)	
Residency	Rural	27(51.9%)	17(42.5%)	0.37
	Urban	25(48.1%)	57.5(23%)	
Family income	Mean (SD)	128.54±41.38	125.13±38.23	0.686
Counselling session attended	Mean(SD)	2.25±.88	3.15±.622	<0.001**

The mean age of the patients chosen for the research was 7.333, with a total of 48 (52.2%) males and 44 (47.8%) females in the study. The compliance group's mean age was found to be considerably higher than the non-compliance group (p=.01). No significant difference was observed in other baseline characteristics such as gender, diagnosis, mental retardation degree, number of siblings, residence location, house ownership, and parents’ education. It was observed that a significant difference was observed between the two groups in the counseling sessions attended. The HAM-A score before pet ownership was comparable before pet ownership (p=.825) but after 3-6 months of pet ownership, a significant difference was observed between compliant and non-compliant groups (p<.001) (Table 2).

**Table 2:** HAM-A score in children pre and post pet ownership *p<0.05; Significant and **p<0.001; Highly Significant HAM-A: Hamilton anxiety scale

	Compliant group	Non-compliant group	p-value
Before pet ownership	19.90±6.6	20.23±7.1	0.825
After 3-6 months of pet ownership	14.44±4.5	20.23±6.9	<0.001**
Change in score between two-time intervals	5.46±3.99	0±1.6	<0.001**
p-value	<0.001**	1	

On comparing the pre and post HAM- A scores of children in the compliant group, a significant difference was observed between the two. On comparing the HAM-A score in patients with mild and moderate mental retardation (mild MR and moderate MR) in the compliant group the significant difference was observed between the two groups at different time interval however no such difference was observed in anxiety levels of children in non-compliant group (Table 3).

**Table 3:** Comparison of HAM-A score between compliant and non-compliant groups with respect to the degree of mental retardation MR: mental retardation *p<0.05; Significant and **p<0.001; Highly Significant

	Degree of retardation(n)	Before pet ownership	After 3-6 months of pet ownership	Change in score	p-value
Compliant group	Mild MR (28)	18.86±7.47	13.79±5.25	5..07±4.21	<0.001**
	Moderate MR (24)	21.13±5.52	15.21±3.40	5.92±3.76	<0.001**
Non -compliant group	Mild MR (20)	19.55±6.29	19.5±5.98	.05±1.57	0.88
	Moderate MR (20)	20.9±8.03	20.9±7.95	-.05±1.76	0.90

It was observed that maximum people 40 (76.9%) had a local dog as their pet followed by a pug, labrador and beagle 3 (5.8%each), dachshund small legs 2 (3.8%), and German shephard 1 (1.9%) It was observed that no significant differences were observed on comparing the HAM-A score with respect to the breed of the pet owned (Table 4).

**Table 4:** Comparison of HAM-A score between children having local dogs and other breeds in the compliant group. *p<0.05; Significant and **p<0.001; Highly Significant

	Desi(local) dogs (n=40)	Others(n=12)	p-value
Pre score	19.98±.3	19.68±8.09	0.89
Post score	14.60±4.72	13.92±3.82	0.65
p-value	<0.001**	0.002*	

The post-ownership score between the children who owned local breed pets and the ones who owned other breeds was comparable (p=0.65).

## Discussion

Many people throughout the globe rely on their pets in their daily lives, and there is mounting evidence linking pet ownership to better health [12,13]. People with anxiety problems may find animal-assisted therapy (AAT) to be a very useful treatment choice. A trained animal may be used in animal therapy to achieve effects that would be impossible to achieve without the animal's presence, and the relationship with the animal makes this possible. Having a dog in the house is not a trivial matter. Over the course of the last 15,000 years, dogs have been safeguarding people by offering a feeling of security and comfort that is not necessarily present in traditional rehabilitation methods [14].

According to Kamioka et al. [15] and Kruger and Serpell [16], animals are a suitable adjunct to anxiety treatment. Seeing a therapist may be stressful for some patients, and having a dog in the room might help ease some of that worry. Using an animal as a distraction is an effective technique to alleviate a patient's fear and anxiety.

Berget and Braastad state that a decrease in anxiety in people with psychiatric disorders is noticeable six months after AAT, but not immediately for the population investigated, still this area of research needs more exploration. Individuals with modest learning difficulties are entitled to the development of innovative psychotherapy procedures that are tailored to their needs. They are aware of what confronts or concerns them in a given existential circumstance, in a more or less frustrating way [17].

In the present study, we sought to evaluate the psychosocial effect of pet ownership in children with mental disabilities. We observed that children who owned pets showed a significant decrease in anxiety levels. Children with both mild and moderate mental disabilities with pet ownership showed significant improvement. We also observed that the decrease in the anxiety levels was comparable in children who owned local breeds and foreign breeds. People who owned local breeds were more at ease because of fewer expenses.

Our study is the first study to assess the anxiety scores in children with mental disabilities. The explanation for the dog's anxiety reduction appears to be due to neurobiological pathways, specifically the brain endocrine system. Oxytocin, a neuropeptide secreted by the hypothalamic supraoptic and paraventricular nuclei, might be held responsible for this decrease [18-20]. As it turns out, oxytocin does have some stress-relieving properties. Low levels of the stress hormone cortisol are associated with high oxytocin levels. Things run well, thanks to these two hormones. Dogs have been shown to have an effect on people's cortisol levels, at least for a short period of time, when they engage pleasantly with them for only a few moments [21].

Despite the fact that dogs have the potential to be an effective therapeutic intervention, dog ownership may benefit some children more than others, and obtaining a dog should only be done with careful consideration [22]. In order to find out which types of impairments are most likely to benefit from owning a dog, further studies are required. Aside from that, the study's biggest flaw is that it's impossible to establish direct comparisons with other research that measure children's anxiety and stress levels.

## Conclusions

This short-term follow-up research highlights the potential advantages of keeping a companion dog for youngsters with mental problems in terms of improving their lives. Many of these long-term gains might be attributed to lessening tensions within families. More high-quality studies on this subject are urgently needed since this research has substantial implications for veterinary, clinical, and family medical practices.
